# The Effect of Thoracolumbar Kyphosis on the Presence and Affected Level of Lumbar Degenerative Spondylolisthesis

**DOI:** 10.3390/jcm15052030

**Published:** 2026-03-06

**Authors:** Şahin Karalar, Muhammed Furkan Darilmaz, Mustafa Abdullah Özdemir, Serkan Bayram, Turgut Akgül, Fatih Dikici

**Affiliations:** 1 Department of Orthopaedics and Traumatology, Faculty of Medicine, İstanbul Medipol University, 34714 Istanbul, Turkey; 2 Training and Research Hospital, Aksaray University, 68100 Aksaray, Turkey; mfdarilmaz@gmail.com; 3 Department of Orthopaedic and Traumatology, Faculty of Medicine, Kahramanmaras Sutcu Imam University, 46050 Kahramanmaras, Turkey; drmustafaozdemir46@gmail.com; 4 Department of Orthopedics and Traumatology, Faculty of Medicine, Istanbul University, 34452 Istanbul, Turkey; dr.serkanbayram89@gmail.com (S.B.); trgtakgul@gmail.com (T.A.); 5 Department of Orthopaedics and Traumatology, Acibadem Maslak Hospital, 34457 Istanbul, Turkey; fatih.dikici@acibadem.edu.tr

**Keywords:** thoracolumbar kyphosis, lumbar degenerative spondylolisthesis, lumbar lordosis, degenerative spine

## Abstract

**Background:** This study aimed to evaluate the relationship between thoracolumbar kyphosis (TLK) and lumbar degenerative spondylolisthesis (LDS) and to determine whether TLK can serve as an independent radiological predictor for both the presence and the specific affected level of LDS. **Methods:** Initially, 211 patients were screened for this study. After applying exclusion criteria, a final cohort of 129 patients (76 women and 53 men; mean age 62.1 ± 9.1 years) who underwent surgical intervention for degenerative lumbar spinal stenosis and had preoperative full-spine standing radiographs were retrospectively analyzed. Patients were divided into two groups: an LDS group (n = 54) comprising patients with concurrent degenerative spondylolisthesis, and a control group (n = 75) consisting of surgical patients without spondylolisthesis. Sagittal parameters, including TLK (T10–L2 angle), pelvic incidence (PI), pelvic tilt (PT), sacral slope (SS), lumbar lordosis (LL), and thoracic kyphosis (TK), were measured. LDS was classified by the affected level (L3–L4, L4–L5, L5–S1). Group differences were compared, ROC analysis was performed to identify a threshold value, and multivariate logistic regression was used to determine independent predictors. **Results:** Multivariate analysis revealed that the T10–L2 angle (TLK) (OR: 1.15, *p* = 0.001), sacral slope (OR: 1.40, *p* = 0.017), pelvic tilt (OR: 1.50, *p* = 0.003), pelvic incidence (OR: 0.68, *p* = 0.004), and lumbar lordosis (OR: 1.09, *p* = 0.005) were significant independent predictors of LDS. Conversely, global thoracic kyphosis (TK) demonstrated an inverse relationship (OR: 0.88, *p* = 0.001), indicative of a secondary compensatory adaptation. ROC analysis established a TLK cut-off of ≥19.5° (AUC = 0.68, *p* = 0.001) for predicting LDS. Furthermore, Roussouly Type 3 alignment was significantly more prevalent in the L5–S1 LDS cohort (48.1%) **Conclusions:** Increased TLK is independently associated with LDS, particularly at lower lumbar levels. A TLK value ≥ 19.5° may serve as a practical radiographic marker, and TLK assessment should be incorporated into sagittal alignment evaluation and surgical planning.

## 1. Introduction

Lumbar degenerative spondylolisthesis (LDS) is a prevalent and debilitating spinal disorder, primarily affecting the elderly population and manifesting with low back pain, radiculopathy, or neurogenic claudication [1]. Numerous studies have investigated its multifactorial etiology, identifying facet joint tropism, disc degeneration, aging, and female sex as significant predisposing factors [2]. Clinically and radiographically, LDS is primarily assessed using standing lateral X-ray examinations, which allow for the dynamic evaluation of vertebral translation and sagittal alignment under physiological weight-bearing conditions [3]. The severity of the vertebral slip is conventionally staged using the Meyerding classification system, which grades the forward translation of the superior vertebra relative to the inferior vertebra from Grade I (0–25% slip) to Grade V (spondyloptosis, >100% slip) [4]. Over the past decade, research has increasingly shifted toward exploring the biomechanical association between spino-pelvic parameters and the development of LDS [5]. Notably, it is now widely accepted that a high pelvic incidence (PI) correlates strongly with the presence of lower lumbar spondylolisthesis, acting as a crucial morphological driver by increasing the shear forces at the lumbosacral junction [6,7].

Beyond isolated pelvic parameters, contemporary spinal research places immense emphasis on global sagittal alignment and its role in various spinal pathologies. As extensively described in the foundational literature, optimal sagittal balance is essential for minimizing energy expenditure and maintaining biomechanical stability [8,9]. When regional malalignment occurs, the spine activates complex compensatory mechanisms, such as pelvic retroversion, to preserve horizontal gaze and an upright posture [10,11]. In this context, Menezes-Reis et al. demonstrated that the pattern of disc degeneration is closely associated with the global sagittal shape of the spine, specifically noting a correlation between the Roussouly type 2 configuration and L4–L5 disc degeneration [10]. Corroborating this, Chung et al. reported that lumbar segmental angles significantly differ among Roussouly spinal types, further linking specific curvature profiles to accelerated segmental wear [12]. These findings suggest that the global sagittal curvature, and particularly its regional transitions, play a critical role in the pathogenesis of LDS [13]. While the relationship between lumbar lordosis and pelvic incidence has been extensively studied, the thoracolumbar junction remains an underappreciated but biomechanically vital area. Roussouly et al. previously described a compensatory pattern involving a long thoracolumbar kyphosis (TLK) paired with short hyperlordosis, which ultimately leads to thoracolumbar discopathy and posterior facet arthritis in the distal lumbar spine [14]. However, despite its established role as a transitional zone subjected to high mechanical stress, the specific relationship between the magnitude of TLK and the exact level of degenerative slips in the lumbar spine remains largely unexplored in the current literature.

Therefore, this study aimed to evaluate the relationship between thoracolumbar kyphosis (TLK) and lumbar degenerative spondylolisthesis (LDS), and to determine whether TLK can serve as an independent radiological predictor for both the presence and the specific affected level of LDS.

## 2. Materials and Method

### 2.1. Research Design and Patient Selection

The study protocol was approved by the Institutional Review Board of Istanbul University Faculty of Medicine (Approval Code: 3183184; Date: 20 January 2025). The requirement for informed consent was waived due to the retrospective design of the study and the use of anonymized radiographic data. A retrospective analysis of prospectively collected data was conducted on an initial cohort of 211 patients who underwent decompression and fusion surgery for degenerative lumbar spinal stenosis between 1 November 2016 and 30 October 2019. Clinical data and radiographic images were evaluated using institutional medical records. After applying the exclusion criteria and assessing the availability of appropriate full-spine standing radiographs, a final cohort of 129 patients was included in the study. The cohort was divided into two distinct groups based on the presence of spondylolisthesis: the LDS group (n = 54) included patients with concurrent lumbar degenerative spondylolisthesis, while the control group (n = 75) consisted of patients with spinal stenosis but without spondylolisthesis. It is crucial to emphasize that although the entire cohort consisted of surgically treated patients, all radiological spinopelvic parameters analyzed in this study were strictly measured from preoperative radiographs. In addition, all patients were classified according to the Roussouly classification system of sagittal spinal alignment. In this system, types 1 and 2 are defined by sacral slope (SS) < 35°, type 3 by SS between 35° and 45°, and type 4 by SS > 45°. Types 1 and 2 generally exhibit low pelvic incidence (PI), whereas types 3 and 4 typically have high PI.

Inclusion criteria were as follows: (1) adult patients (aged > 18 years) with a history of chronic low back pain; (2) presence of lumbar degenerative spondylolisthesis (for the study group) or absence of LDS (for the control group); (3) availability of high-quality, preoperative standing full-spine radiographs; and (4) availability of complete demographic data and medical records.

Exclusion criteria included: (1) history of spinal trauma, tumor, or infection; (2) previous spinal surgery; (3) congenital spinal deformities; (4) coronal deformity (scoliosis) with a Cobb angle greater than 10°; (5) history of hip or knee arthroplasty; (6) incomplete medical records; (7) dysplastic, isthmic, or traumatic spondylolisthesis; and (8) patients who required knee flexion to compensate for sagittal malalignment.

All patients in the LDS group presented with low-grade degenerative spondylolisthesis (Meyerding Grade I or II). A subgroup analysis based on the specific degree of the vertebral slip was not performed, as the primary objective of this study was to evaluate the relationship between sagittal parameters and the specific affected level of the listhesis.

Surgical indication for the patients in the LDS group was based on the presence of chronic and persistent pain refractory to conservative treatments, neurological deficits, or significant sagittal imbalance. The surgical strategy was meticulously tailored to the patient’s specific pathoanatomy and spinal stability. Decompression alone was performed in patients without radiographic instability and with preserved sagittal alignment. Conversely, in cases presenting with radiological evidence of instability and sagittal malalignment, posterior neural decompression was combined with instrumented fusion. For these fusion procedures, interbody fusion techniques—such as transforaminal (TLIF), posterior (PLIF), or anterior lumbar interbody fusion approaches—were utilized, incorporating titanium interbody cages in select cases. Standard titanium pedicle screw-rod systems were uniformly used for posterior instrumentation across all fusion cases. It is important to emphasize that while all patients in the LDS group ultimately required surgery, the primary focus of this study was the analysis of preoperative spinal alignment; therefore, all radiographic measurements were strictly obtained from images acquired prior to any surgical intervention.

### 2.2. Radiological Measurement

All radiological evaluations were performed using standardized standing full-spine lateral radiographs. During the examination, patients were instructed to stand in a relaxed, natural posture with their hips and knees fully extended. To provide a clear view of the spine without upper extremity interference, patients were asked to rest their fists on their clavicles. Each lateral radiograph included the entire pelvis and extended cranially to include the C7 vertebra. All sagittal radiographic parameters were independently and digitally measured using Surgimap software (Version 2.3.2.1, Nemaris Inc., New York, NY, USA). All radiographic parameters were independently measured by two orthopedic surgeons. Interobserver reliability was assessed using the intraclass correlation coefficient (ICC), which demonstrated excellent agreement across all measurements (ICC range: 0.88–0.94). The sagittal spinopelvic parameters were measured according to the standardized techniques established in the literature [15,16]. Specifically, the pelvic parameters—pelvic incidence (PI), pelvic tilt (PT), and sacral slope (SS)—were measured using the foundational principles described by Legaye and Duval-Beaupère [17]. Lumbar lordosis (LL) and thoracic kyphosis (TK) were assessed using the standard Cobb method [18]. Furthermore, thoracolumbar kyphosis (TLK), defined as the angle between the superior endplate of T10 and the inferior endplate of L2, was measured in accordance with standard sagittal alignment protocols [9].

**Figure 1:**
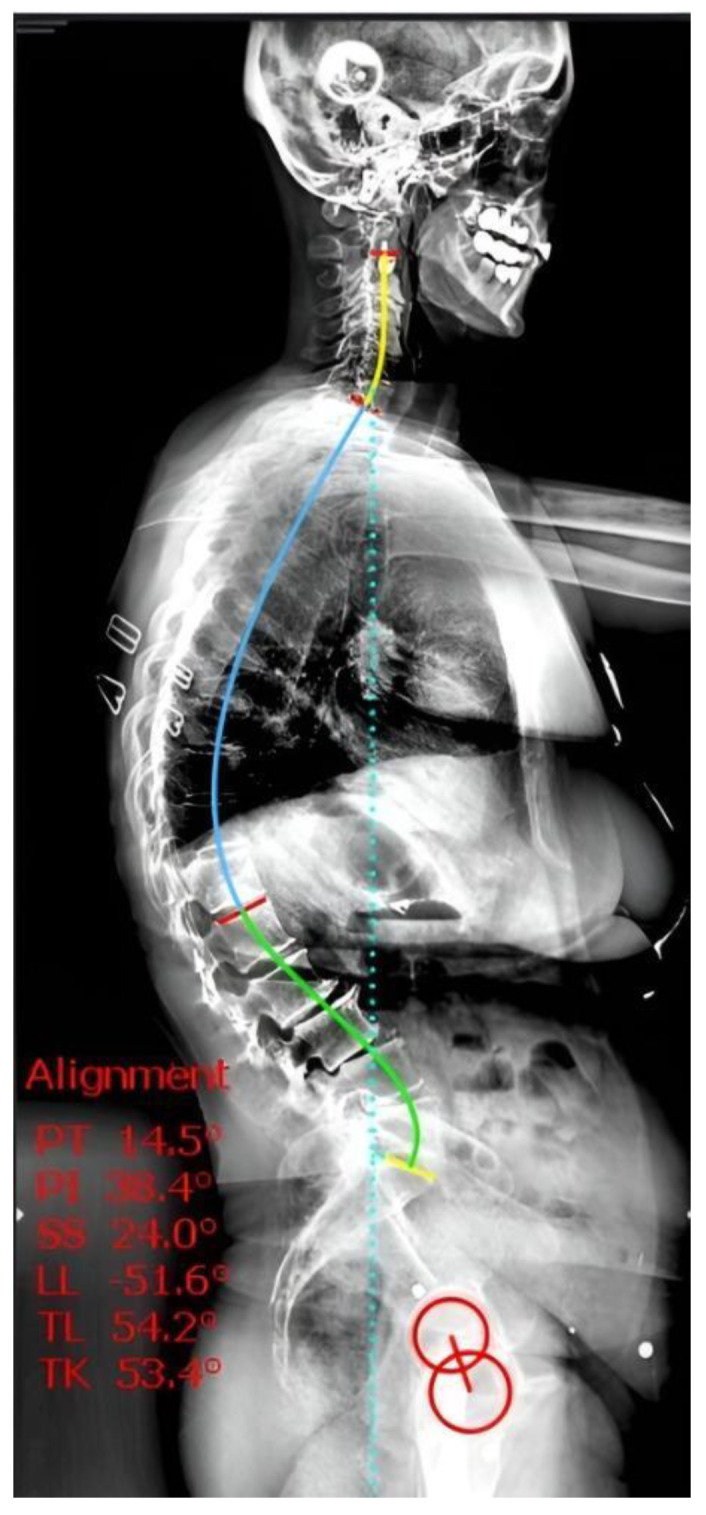
Demonstrative full-spine lateral radiograph illustrating the radiological measurement methods used in this study. The sagittal spinopelvic parameters, including Thoracolumbar Kyphosis (TL, measured as the T10–L2 angle), Lumbar Lordosis (LL), Pelvic Incidence (PI), Pelvic Tilt (PT), and Sacral Slope (SS), were digitally measured using Surgimap software (Version 2.3.2.1, Nemaris Inc., New York, NY, USA).

The following sagittal alignment parameters were measured (Figure 1):

- Sagittal vertical axis (SVA): the distance between the C7 plumb line and the posterior superior corner of the sacrum, reflecting anterior trunk inclination.
- Thoracic kyphosis (TK): angle between the upper endplate of T4 and the upper endplate of T12.
- Lumbar lordosis (LL): angle between the upper endplate of L1 and the upper endplate of S1.
- Thoracolumbar kyphosis (TLK): angle between the upper endplate of T10 and the upper endplate of L2.
- Sacral slope (SS): angle between the superior endplate of the sacrum and the horizontal line.
- Pelvic tilt (PT): angle between the vertical line and the line connecting the midpoint of the sacral plate to the center of the femoral heads.
- Pelvic incidence (PI): angle between the perpendicular to the sacral plate at its midpoint and the line connecting this point to the midpoint of the femoral heads.
- T1 pelvic angle (TPA): angle between the line from the femoral head axis to the centroid of the T1 vertebral body and the line from the femoral head axis to the center of the S1 endplate.
- T1 spinopelvic inclination (T1SPI): angle between the vertical line and the line connecting the centroid of T1 to the bicoxofemoral axis.
- T9 spinopelvic inclination (T9SPI): angle between the vertical line and the line connecting the centroid of T9 to the bicoxofemoral axis.

### 2.3. Statistical Analyses

All statistical analyses were performed using IBM SPSS Statistics for Windows, Version 24.0 (IBM Corp., Armonk, NY, USA). Descriptive statistics were used to summarize the study data. The normality of distribution for continuous variables was assessed using the Shapiro–Wilk test. For normally distributed variables, Pearson correlation analysis was used to evaluate the relationships between parameters; for non-normally distributed variables, Spearman rank correlation analysis was applied. A *p*-value < 0.05 was considered statistically significant. A receiver operating characteristic (ROC) curve analysis was conducted to determine the optimal cut-off value of thoracolumbar kyphosis for predicting lumbar degenerative spondylolisthesis. The ROC curve was used to estimate the cut-off value of thoracolumbar kyphosis. AUC values were calculated with 95% confidence intervals.

## 3. Results

A total of 129 patients (76 women and 53 men) with a mean age of 62.1 ± 9.1 years (range, 38–79 years) who underwent surgical intervention for degenerative lumbar spinal stenosis were included in the final analysis. Within this surgical cohort, 75 patients had no evidence of lumbar degenerative spondylolisthesis (Control group), whereas 54 patients presented with concurrent LDS (LDS group). In the control group, there were 46 women and 29 men, while the LDS group consisted of 30 women and 24 men. There was no statistically significant difference between the groups in terms of gender distribution (*p* = 0.634). The distribution of LDS was as follows: 7 patients at L3–L4, 18 patients at L4–L5, and 27 patients at L5–S1 levels; one patient had LDS at two levels. Baseline demographic characteristics and radiographic parameters of each group are summarized in Table 1.

**Figure 2:**
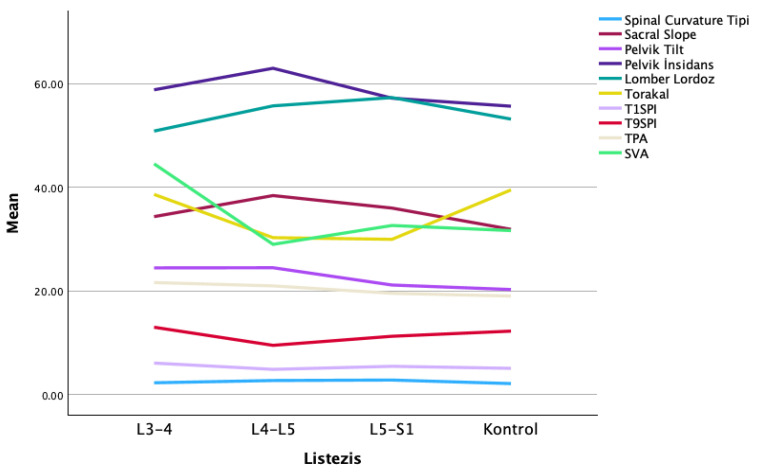
The analysis of spinopelvic parameters revealed level-specific variations among the lumbar degenerative spondylolisthesis (LDS) groups.

The analysis of spinopelvic parameters revealed distinct, level-specific variations across the lumbar degenerative spondylolisthesis (LDS) groups (Figure 2). Pelvic incidence (PI) and sacral slope (SS) reached their peak values in the L4–L5 listhesis group. Conversely, the sagittal vertical axis (SVA) showed a substantial increase within the L3–L4 group when compared to both the L5–S1 and control groups. Pelvic tilt (PT) was also observed to be relatively higher in the L3–L4 and L4–L5 groups. Furthermore, a progressive increase in lumbar lordosis (LL) toward the L5–S1 level was accompanied by an inversely proportional decrease in thoracic kyphosis (TK).

**Figure 3:**
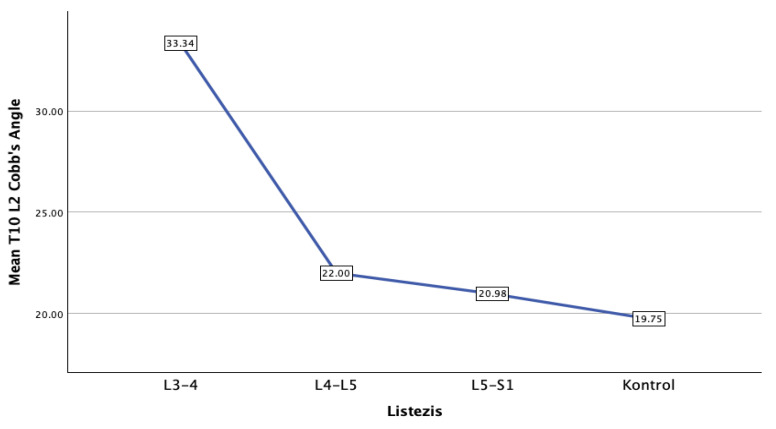
Illustrates the mean thoracolumbar kyphosis (TLK, measured as the T10–L2 angle) across the different lumbar degenerative spondylolisthesis (LDS) levels and the control group. The data reveals a distinct anatomical trend: the highest mean TLK value was observed in patients with L3–L4 spondylolisthesis (33.34°). This value sharply decreases in the lower lumbar segments, presenting as 22.00° in the L4–L5 group and 20.98° in the L5–S1 group. The control group exhibited the lowest overall mean TLK at 19.75°. These findings demonstrate a pronounced increase in thoracolumbar kyphosis specifically associated with upper lumbar (L3–L4) degenerative slips compared to lower levels and healthy individuals.

Thoracolumbar kyphosis, as measured by the T10–L2 angle, differed significantly between LDS and control groups. The highest mean TLK was observed in the L3–L4 LDS group (33.34° ± 16.02°), compared to lower values in L4–L5 (22.00° ± 4.12°), L5–S1 (20.97° ± 4.37°), and the control group (21.34° ± 8.09°) (*p* = 0.001). Multiple comparison tests confirmed that the L3–L4 group had significantly greater TLK compared to all other groups (Figure 3).

**Figure 4:**
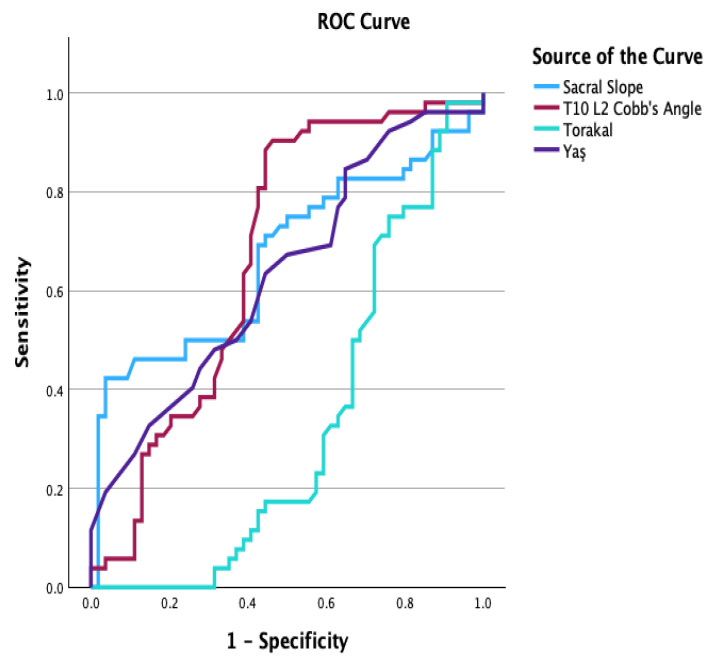
Receiver Operating Characteristic (ROC) curves evaluating the predictive value of demographic and radiological parameters (T10–L2 angle, Sacral Slope, Thoracic Kyphosis, and Age) for lumbar degenerative spondylolisthesis.

To evaluate the predictive value and determine the optimal cut-off thresholds of continuous demographic and radiological parameters for lumbar degenerative spondylolisthesis, a Receiver Operating Characteristic (ROC) curve analysis was conducted. The analysis revealed that a T10–L2 angle of 19.5° or greater was a significant predictor, yielding an area under the curve (AUC) of 0.68 (sensitivity: 64%, specificity: 62%; *p* = 0.001). Similarly, a sacral slope cut-off value of 33.6° demonstrated an AUC of 0.68 (sensitivity: 58%, specificity: 58%; *p* = 0.002). Age was also identified as a significant predictive factor, with an optimal cut-off of 64.5 years (AUC: 0.64, sensitivity: 64%, specificity: 56%; *p* = 0.012). Furthermore, thoracic kyphosis yielded an AUC of 0.34 (cut-off: 33.95°, sensitivity: 37%, specificity: 36%; *p* = 0.001). An AUC value below 0.5 for thoracic kyphosis indicates a significant inverse relationship, corroborating our regression findings that lower thoracic kyphosis values are strongly associated with the presence of spondylolisthesis. ROC findings are presented in Table 2 and illustrated in Figure 4.

To identify the independent risk factors associated with LDS, univariate and multivariate logistic regression analyses were performed (Table 3). In the univariate analysis, age (OR: 1.06, *p* = 0.018), T10–L2 angle (OR: 1.06, *p* = 0.048), sacral slope (OR: 1.07, *p* = 0.006), positive shift in sagittal vertical axis (SVA) (OR: 2.37, *p* = 0.003), and Type 3 spinal curvature (OR: 7.05, *p* = 0.003) were found to be significantly associated with an increased likelihood of LDS. Conversely, thoracic kyphosis (OR: 0.95, *p* = 0.002) demonstrated a significant protective effect.

A subsequent multivariate logistic regression model was constructed to adjust for potential confounding variables. The multivariate analysis revealed that T10–L2 angle (OR: 1.15, 95% CI: 1.06–1.25; *p* = 0.001), sacral slope (OR: 1.40, 95% CI: 1.06–1.85; *p* = 0.017), and thoracic kyphosis (OR: 0.88, 95% CI: 0.82–0.95; *p* = 0.001) maintained their statistical significance as independent predictors. Notably, while pelvic tilt, pelvic incidence, and lumbar lordosis did not exhibit statistical significance in the initial univariate assessment, they emerged as significant independent predictors in the multivariate model (Pelvic tilt: OR: 1.50, 95% CI: 1.15–1.96, *p* = 0.003; Pelvic incidence: OR: 0.68, 95% CI: 0.53–0.88, *p* = 0.004; Lumbar lordosis: OR: 1.09, 95% CI: 1.02–1.15, *p* = 0.005). Furthermore, variables such as age, SVA, and Type 3 spinal curvature lost their statistical significance in the multivariate model (*p* > 0.05).

Radiological parameters and the distribution of lumbar degenerative spondylolisthesis (LDS) were further stratified according to the Roussouly spinal curvature classification (Types I–IV). A significant difference in the distribution of these curvature types was observed between the LDS and control groups (*p* = 0.027, Chi-square test). Specifically, Roussouly Types I and II were predominantly found in the control group (Type I: 35.2%), whereas Type III was significantly more prevalent in the L5–S1 LDS cohort (48.1%). This indicates that a Type III sagittal profile may predispose individuals to distal-level spondylolisthesis. Detailed comparisons are presented in Table 4.

## 4. Discussion

The principal finding of the present study is the significant association between increased thoracolumbar kyphosis (TLK) and the presence of lumbar degenerative spondylolisthesis (LDS). The results demonstrated that patients with LDS exhibited significantly greater TLK compared to those without LDS (*p* < 0.001, r = 0.609). This strongly supports the hypothesis that sagittal spinal alignment, particularly in the thoracolumbar region, plays a pivotal role in the pathogenesis of LDS. These findings are highly consistent with previous literature emphasizing the critical influence of sagittal balance and spinopelvic morphology on lumbar spine degeneration. Notably, Kobayashi et al. demonstrated that global sagittal spinal alignment is significantly altered in patients with degenerative low-grade lumbar spondylolisthesis [19]. Similarly, Nakamae et al. reported a strong correlation between sagittal spinopelvic malalignment and the severity of LDS, highlighting how altered biomechanical forces contribute to segmental instability [20]. In accordance with these studies, our results further reinforce the concept that regional sagittal alignment changes, such as increased TLK, act as critical mechanical contributors to the development of LDS.

The findings of the present study demonstrate that the heterogeneous, level-specific biomechanical effects observed in this cohort indicate that the level of spondylolisthesis distinctly alters spinopelvic alignment. The peak PI and SS values in the L4–L5 group support the premise that high pelvic incidence may serve as a predisposing morphological factor for pathogenesis specifically at this level. Conversely, the substantial increase in SVA—a key indicator of global sagittal balance—within the L3–L4 group indicates that listhesis at upper lumbar levels disrupts global alignment significantly more severely than at lower levels. The regional parameter variations reflect the distinct compensatory mechanisms recruited to restore this impaired balance. The higher PT observed in the L3–L4 and L4–L5 groups reflects an active effort to compensate for the anteriorly shifted sagittal balance through pelvic retroversion. Meanwhile, the regional adaptations of the spine are further revealed by the progressive increase in LL and the corresponding decrease in TK. Ultimately, the characterization of L4–L5 listhesis by high anatomical predisposition, alongside L3–L4 listhesis being marked by profoundly impaired global sagittal balance, underscores the critical importance of incorporating level-specific spinopelvic dynamics and compensatory mechanisms into clinical evaluations and surgical planning. Prior literature has predominantly focused on classical spinopelvic parameters such as pelvic incidence (PI), pelvic tilt (PT), sacral slope (SS), and lumbar lordosis (LL) in the context of lumbar spine degeneration. For instance, Barrey et al. demonstrated that patients with lumbar degenerative spondylolisthesis (LDS) exhibit decreased LL alongside increased PT and PI, highlighting spinopelvic imbalance as a major biomechanical contributor to the condition [21]. More recently, Singh et al. reaffirmed the critical role of sagittal alignment, emphasizing how variations in these spino-pelvic parameters alter the mechanical environment of the aging spine and predispose specific lumbar segments to olisthesis [22]. Similarly, Labelle et al. previously identified elevated LL and PT as factors closely associated with increased slip severity. Our findings partially corroborate these established paradigms; we observed modest but significant correlations between LDS at the L4–L5 level and both PT (*p* = 0.026, r = 0.196) and PI (*p* = 0.045, r = 0.177) [23]. However, contrary to some of the aforementioned reports, we found no significant bivariate correlation between overall LL and the presence of LDS. This discrepancy suggests that while traditional pelvic parameters remain relevant, examining regional dynamic changes—such as thoracolumbar curvature—may offer superior predictive insight into the pathogenesis of LDS than relying solely on lumbar lordosis.

Furthermore, the differential impact of regional sagittal parameters is highlighted by the findings regarding overall thoracic kyphosis (TK). While an increased T10–L2 angle acts as a risk factor for LDS, the multivariate analysis revealed an inverse relationship between global TK and the presence of LDS (OR: 0.88, *p* = 0.001). This seemingly paradoxical finding can be best explained by the global compensatory mechanisms of the aging spine. As distal lumbar segments become unstable and lose their natural lordosis due to degeneration and slippage, the trunk tends to lean forward, causing a positive shift in the sagittal vertical axis (SVA). To counteract this forward imbalance and maintain the center of gravity over the pelvis, the body dynamically recruits compensatory mechanisms, one of the most prominent being the reciprocal flattening of the thoracic spine (thoracic hypokyphosis) [24,25]. Previous studies by Schwab et al. have extensively described these postural adaptations, noting that a reduction in TK often occurs secondary to lower lumbar pathology to preserve horizontal gaze and global alignment [8]. Therefore, the reduced TK observed in our LDS cohort is likely a secondary compensatory adaptation rather than a primary pathogenic driver. In contrast, the localized angular changes at the thoracolumbar junction (TLK) directly contribute to the adverse mechanical shear forces that precipitate the spondylolisthesis.

Receiver operating characteristic (ROC) curve analysis determined that a TLK value of 19.5° represents the optimal cut-off for predicting LDS, corresponding to a sensitivity of 64%, a specificity of 62%, and an area under the curve (AUC) of 0.68. Although the observed discriminative performance is within the moderate range, these findings suggest that TLK may serve as a clinically relevant screening parameter. Notably, to the best of our knowledge, no previous study has established a quantitative TLK threshold for the prediction of LDS, thereby emphasizing the originality and potential clinical relevance of the present results.

Spinopelvic and global sagittal alignment discrepancies between LDS and non-LDS patients have been well documented [8,20,21,22,23,24,25,26]. Funao et al. showed that LDS patients have higher PI, SS, LL, TK, and lower slope angles compared to non-LDS individuals [26]. Nakamae et al. found strong correlations between LDS severity and PI, SS, and LL, particularly at L4 and L5 levels [27]. However, their findings did not highlight TLK as a contributory factor. In contrast, the current results suggest that TLK may act as an independent contributor to the anterior shear forces responsible for spondylolisthesis, particularly at lower lumbar levels. The increased TLK may reflect a compensatory shift in sagittal balance that predisposes to slip progression.

This study has several limitations. First, it is a retrospective, single-center analysis with a relatively limited sample size. The LDS cohort was not stratified by Meyerding grades or categorized into anterior versus posterior slips, which may have obscured specific alignment-related nuances. Furthermore, other pertinent morphological factors—such as disc height, facet joint angulation, or vertebral body shape—were not evaluated. A notable limitation is the specific inclusion of patients who ultimately underwent surgical intervention for LDS. While this strategy ensured the availability of high-quality, standardized preoperative full-spine standing radiographs (crucial for accurate spino-pelvic measurements), it inevitably introduces selection bias. Our cohort primarily represents symptomatic individuals with advanced degeneration requiring decompression and fusion; therefore, these findings may not fully extrapolate to asymptomatic individuals or those managed conservatively. Importantly, because all measurements were strictly derived from preoperative images, the surgical procedures did not confound the analyzed sagittal alignment.

Despite these limitations, identifying thoracolumbar kyphosis (TLK) as a novel and independent predictor of LDS contributes meaningfully to the literature and opens several avenues for future research. Prospective, longitudinal studies are warranted to establish definitive causality—specifically, to ascertain whether increased TLK is a primary predisposing morphological factor for lower lumbar LDS or a secondary compensatory mechanism. Additionally, future investigations should incorporate postoperative radiological and clinical outcomes to evaluate whether surgical restoration of TLK to physiological levels (e.g., via osteotomies) yields superior long-term results compared to isolated short-segment fixation. The integration of advanced imaging modalities, such as 3D stereoradiography or magnetic resonance imaging (MRI) for assessing paraspinal muscle quality, would also provide a more comprehensive biomechanical profile. Finally, large-scale, multicenter studies are essential to validate the proposed TLK threshold of 19.5° and refine surgical decision-making algorithms.

## 5. Conclusions

This study identifies increased thoracolumbar kyphosis (TLK) as a novel and independent predictor for the development of lower lumbar degenerative spondylolisthesis (LDS), particularly at the L4–L5 and L5–S1 levels. A T10–L2 angle greater than 19.5° significantly alters the biomechanical environment, increasing anterior shear forces at the distal lumbar segments. Furthermore, the observed reduction in global thoracic kyphosis highlights the dynamic compensatory mechanisms of the aging spine in response to sagittal imbalance. For clinical practice, it is imperative that spine surgeons routinely incorporate the assessment of regional thoracolumbar alignment into preoperative planning. Recognizing pathological TLK can guide more precise surgical decision-making, optimize alignment restoration goals, and potentially mitigate the risk of postoperative complications such as adjacent segment disease and mechanical failure.

## Figures and Tables

**Table 1:** Demographic and preoperative radiological characteristics of the surgical cohort, stratified by the presence of lumbar degenerative spondylolisthesis (LDS).

Parameter	L3–L4 LDS (n = 7)	L4–L5 LDS (n = 18)	L5–S1 LDS (n = 27)	Control Group (n = 75)	*p*-Value
Age (years)	64.00 ± 8.40	65.61 ± 9.21	68.07 ± 7.47	64.90 ± 7.39	0.042 *
Sex (F/M)	30/24			46/29	0.634
Sacral Slope (°)	34.32 ± 12.00	38.38 ± 9.82	35.97 ± 8.81	34.18 ± 8.60	0.023 *
Pelvic Tilt (°)	24.44 ± 16.02	24.47 ± 10.04	21.13 ± 10.63	21.48 ± 10.55	0.439
Pelvic Incidence (°)	58.78 ± 26.90	62.93 ± 12.50	57.17 ± 12.74	57.46 ± 16.03	0.417
Lumbar Lordosis (°)	50.78 ± 12.05	55.68 ± 16.62	54.32 ± 16.52	54.47 ± 16.11	0.653
T10–L2 Angle (°)	33.34 ± 16.02	22.00 ± 4.12	20.97 ± 4.37	21.34 ± 8.09	0.001 *
Thoracic Kyphosis (°)	38.61 ± 9.14	30.27 ± 11.98	29.95 ± 14.06	35.43 ± 13.37	0.005 *
T1SPI (°)	6.08 ± 3.69	4.87 ± 4.58	5.46 ± 3.54	5.20 ± 3.68	0.865
T9SPI (°)	13.00 ± 8.32	9.50 ± 5.37	11.25 ± 5.67	11.57 ± 5.73	0.310
TPA (°)	21.61 ± 16.88	20.97 ± 12.40	19.54 ± 12.57	19.64 ± 15.17	0.951
SVA (mm)	44.51 ± 31.98	28.98 ± 23.07	32.61 ± 31.15	32.28 ± 29.81	0.705

(LDS: Lumbar Degenerative Spondylolisthesis. All patients underwent surgical intervention for degenerative lumbar spinal stenosis. The Control Group consists of patients without concurrent LDS.). * indicates statistical significance (*p* < 0.05).

**Table 2:** ROC Analysis for Predicting Lumbar Degenerative Spondylolisthesis.

Parameter	Cut-off	Sensitivity (%)	Specificity (%)	AUC	*p*-Value
T10–L2 Cobb’s Angle	19.5°	64	62	0.68	0.001 *
Sacral Slope	33.6°	58	58	0.68	0.002 *
Thoracic Kyphosis	33.95°	37	36	0.34	0.001 *
Age	64.5	64	56	0.64	0.012 *

* indicates statistical significance (*p* < 0.05). AUC: Area Under the Curve.

**Table 3:** Univariate and Multivariate Logistic Regression Analysis.

Parameter	OR (Univariate)	*p*-Value	OR (Multivariate)	*p*-Value
T10–L2 Cobb’s Angle	1.06 (1.00–1.12)	0.048 *	1.15 (1.06–1.25)	0.001 *
Sacral Slope	1.07 (1.02–1.12)	0.006 *	1.40 (1.06–1.85)	0.017 *
Pelvic Tilt	1.02 (0.98–1.06)	0.230	1.50 (1.15–1.96)	0.003 *
Pelvic Incidence	1.01 (0.99–1.04)	0.228	0.68 (0.53–0.88)	0.004 *
Lumbar Lordosis	1.01 (0.98–1.03)	0.384	1.09 (1.02–1.15)	0.005 *
Thoracic Kyphosis	0.95 (0.91–0.99)	0.002 *	0.88 (0.82–0.95)	0.001 *
Age	1.06 (1.01–1.13)	0.018 *	1.04 (0.97–1.13)	0.231
Spinal Curvature Type (Type 3)	7.05 (1.83–27.14)	0.004 *	5.89 (0.25–134.0)	0.266
SVA (Positive shift)	2.37 (0.88–6.38)	0.048 *	1.78 (0.37–8.53)	0.468

Data are presented as Odds Ratios (OR) with 95% Confidence Intervals (CI) in parentheses. Univariate and multivariate logistic regression analyses were performed to identify independent risk factors. * indicates statistical significance (*p* < 0.05).

**Table 4:** Distribution of Roussouly Spinal Curvature Types and Radiological Parameters.

Spinal Curvature Type	L3–L4 (%)	L4–L5 (%)	L5–S1 (%)	Control (%)	Chi-Square *p*-Value
Type 1	28.6	22.2	3.7	35.2	0.027 *
Type 2	42.9	16.7	29.6	25.9	
Type 3	0.0	27.8	48.1	29.6	
Type 4	28.6	33.3	18.5	9.3	

(LDS: Lumbar Degenerative Spondylolisthesis. All patients underwent surgical intervention for degenerative lumbar spinal stenosis. The Control Group consists of patients without concurrent LDS.). Data are presented as percentages (%) for the categorical variables. The distribution of Roussouly spinal curvature types among the groups was evaluated using the Chi-Square test. * indicates statistical significance (*p* < 0.05).

## Data Availability

The data presented in this study are available on request from the corresponding author. The data are not publicly available due to privacy and ethical restrictions.

## Abbreviations

TLK	Thoracolumbar Kyphosis
LDS	Lumbar Degenerative Spondylolisthesis
TK	Thoracic Kyphosis
LL	Lumbar Lordosis
PI	Pelvic Incidence
PT	Pelvic Tilt
SS	Sacral Slope
SVA	Sagittal Vertical Axis
TPA	T1 Pelvic Angle
T1SPI	T1 Spinopelvic Inclination
T9SPI	T9 Spinopelvic Inclination
ICC	Intraclass Correlation Coefficient
ROC	Receiver Operating Characteristic
AUC	Area Under the Curve
OR	Odds Ratio
CI	Confidence Interval
SPSS	Statistical Package for the Social Sciences
